# Associations of muscle-strengthening exercise with overweight, obesity, and depressive symptoms in adolescents: Findings from 2019 Youth Risk Behavior Surveillance system

**DOI:** 10.3389/fpsyg.2022.980076

**Published:** 2022-09-08

**Authors:** Jizu Shi, Mingjun Gao, Xiao Xu, Xuyang Zhang, Jin Yan

**Affiliations:** ^1^Key Laboratory of Endurance Sport, Jilin Sport University, Changchun, China; ^2^Foundation Department of Education, Shandong Communication and Media College, Jinan, China; ^3^China Basketball College, Beijing Sport University, Beijing, China; ^4^Centre for Active Living and Learning, University of Newcastle, Callaghan, NSW, Australia; ^5^College of Human and Social Futures, University of Newcastle, Callaghan, NSW, Australia

**Keywords:** muscle-strengthening exercise, body mass index, high school students, the Youth Risk Behavior Surveillance, overweight

## Abstract

**Background:**

Previous studies have focused on the opposite relation between muscle strength, obesity, and depression in adults. Moreover, the evidence has indicated that obesity and depression in adults might be significantly decreased with Muscle Strength Exercise (MSE) albeit it might be insufficient. Therefore, the current study aimed to investigate the association between MSE, adiposity, and depression among United States adolescents.

**Materials and methods:**

This cross-sectional study used the Youth Risk Behavioral Survey (YRBS) data. In YRBS, a cluster sample was used, and the investigation was divided into three stages. The study surveyed 13,677 high school students and conducted self-reported questionnaires on sex, grade, race/ethnicity, MSE days, overweight, obesity, and depressive symptoms. The study got the nationally representative population of American students in Grade 9 to 12 (around 12–18 years).

**Results:**

A total of 13,677 participants (female = 6,885, male = 6641) were included in the final analysis. The participants meeting the guidelines’ requirements seemed more likely to be obese than those not meeting (OR = 1.28, 95% CI = 1.06–1.55). There was no statistical significance in the relations between the MSE guidelines and overweight and depression (OR = 0.86, 95% CI = 0.73–1.01: OR = 0.94, 95% CI = 0.83–1.06). For all the participants, the prevalence of those conforming to MSE was 30.1%. One-fifth of the participants reported no MSE per week, 7.8% reported 3 days of MSE per week, and 7.7% reported 7 days.

**Conclusion:**

The main finding of this study indicated a positive relationship between the normative MSE required in guidelines and low-level obesity. Beyond that, the evidence was insufficient to confirm the positive links between MSE and depression among American adolescents. Our study could offer evidence for future MSE interventions in adolescents.

## Introduction

Overweight and obesity are excessive or abnormal fat accumulation that is risky to people’s health (Abdelaal et al., 2017; Piché et al., 2020). A person is overweight if the Body Mass Index (BMI) exceeds a certain value. People are obese if their BMI exceeds 30 (Ho-Pham et al., 2015; World Health Organization, 2021). Obesity is a critical risk factor for mortality and morbidity, as it also accounts for a life expectancy drop (Abdelaal et al., 2017; Blüher, 2019). For example, according to past research, there is an association between obesity and metabolic diseases (fatty liver disease and type 2 diabetes), cardiovascular diseases such as stroke, myocardial infarction, and hypertension, mental health such as self-esteem and depression, musculoskeletal diseases, bullying, and some cancers (Daniels et al., 2009; Osier et al., 2017). In the past 50 years, the prevalence of obesity has been high globally. For instance, from 1975 to 2016, the overweight prevalence increased significantly, considering the number of obese adolescents and children aged between 5 and 18 years increased by four times, from 4 to 18% worldwide (Suder et al., 2017; Magriplis et al., 2021; World Health Organization, 2021). Similarly, the results of some research indicated that the trend of increased obesity cases was identified among adolescents in the US, and the problem of obesity might lead to medical expenditures of up to 300 billion dollars every year (Kosti and Panagiotakos, 2006; Hammond and Levine, 2010).

Considering the negative impacts of obesity, reducing the prevalence of obesity among adolescents has become a high priority for most countries. The World Health Organization (WHO) recommended that children and adolescents aged 5–17 years should participate in vigorous-intensity aerobic physical activities and Muscle Strength Exercise (MSE) at least 3 days a week (Foster et al., 2018; Bull et al., 2020; Lin and Yan, 2020; World Health Organization, 2020; Gu et al., 2022; Shi et al., 2022). Several studies revealed that physical activity intervention combined with a nutritional component was a major effective way to decrease obesity and overweight among children and adolescents (Kelley and Kelley, 2013; Mei et al., 2016; Godoy-Cumillaf et al., 2019, 2020). Other studies indicated that traditional physical activity approaches might exert small effects on preventing youth obesity (Guerra et al., 2014; Peirson et al., 2015). Besides, one study showed that aerobic training intervention had small effects on reducing body fat and body mass percentage among adolescents (Schranz et al., 2013). Although regular aerobic moderate-to-vigorous physical activities are of great health significance (Shen et al., 2020; Chen et al., 2022), MSE can be an alternative form of physical activities for those people who have barriers to perform traditional physical activities (e.g., adolescents with disabilities) (Bennie et al., 2020a).

Muscle strength exercise can be defined as a voluntary strength/weight/resistance activity that includes using weight machines, exercise bands, hand-held weights, or own body weight (Ratamess et al., 2009; Lin and Yan, 2020). MSE is beneficial to increasing muscle strength and endurance, aerobic fitness, and bone mineral density, improving body composition, and obtaining lower body pressure among adolescents (Faigenbaum and Myer, 2010; Chen et al., 2022; Gu et al., 2022). According to emerging epidemiological evidence, muscle strength was inversely associated with obesity (Thivel et al., 2016) and a lower level of cardiovascular disease risk factors (Grøntved et al., 2015) among young people. For instance, a cross-sectional study conducted among children and adolescents indicated that overweight or obesity was linked to a lower level of handgrip strength among boys and girls (Palacio-Agüero et al., 2020).

Early studies have suggested the association between individuals’ physical characteristics and mood disorders. In the longitudinal research conducted by Okholm et al. (2021), the researchers recruited 630,807 male participants from Denmark. After following the participants for 26 years and collecting relevant information about their physical activities and health data, the study suggested that individuals with lower BMI have lower risks of mood disorders as they become adults. Nevertheless, this study’s sample only involved Danish men aged from 18 to 24 years old, and the findings might not be generalizable to a larger population such as American adolescents. Also, Bao et al. (2022) conducted a 7-year longitudinal study to investigate the relationship between individuals’ muscle strength and the risk of depression. As the findings suggested, an individual’s muscle mass developed a significant negative association with the development of depressive symptoms. Furthermore, the study demonstrated that lack of muscle mass might be a predictive risk factor for depression among middle-aged and elderly individuals (Bao et al., 2022). However, the impact of MSE on depression remained unclear.

Moreover, a significant association between physical activities and depressive symptoms among adults over 45 years old has been found. In a newly published study conducted by Kim (2022), it has been suggested that individuals’ daily exercise time duration might be negatively linked with symptoms of depression, suggesting the preventive effect of regular exercise on depression. However, the participants of this study were adults over 45 years old, and this finding might not be generalizable to the younger adolescent population.

Previous studies investigated the relationship between daily exercise levels and depression. Little evidence has been found to demonstrate the association between MSE and individuals’ depressive symptoms, especially for this kind of sample based on the national representative. Therefore, this cross-sectional study targeted identifying the association between MSE, adiposity, and depression among American adolescents.

## Materials and methods

### Study population

This study used the 2019 Youth Risk Behavior Surveillance System data (YRBS), a national survey conducted by the Centers for Disease Control and Prevention (CDC). More details on the YRBS can be found through the link.^1^ In 2019, a self-administered survey of YRBS was conducted, and the survey involved 13,677 students of high schools based on a cluster-sample design of three stages. The population with national representativeness is obtained from the students in grades 9 to 12 in the United States. The general response rate is 60%. Based on the YRBS sample, estimates are obtained with accuracy within ± 5% at a 95% confidence level.

### Overweight/obesity

Within the YRBS, the determination of overweight and obesity was derived from body mass index (BMI) percentiles. Self-reported height and weight of study participants were collected and converted into BMI for classifying overweight or obesity according to CDC BMI age- and sex-specific percentiles (BMI was ≥ 85th percentile). In sum, of the total eligible study participants, 27.2% of them were classified as overweight or obese.

### Muscle-strengthening exercise

It was required for participants to submit their information on MSE. The question was “How many MSEs did you do last week to build muscle?” MSE referred to activities involving major muscle groups in this study, such as push-ups, weightlifting, crunches, or pull-ups, and the definition can offer assistance to participants in understanding and filling out questionnaires. Participants’ response times ranged from 0 to 7 days. This method was taken as a reliable and effective method to evaluate the MSE of young people (Song et al., 2013; Gu et al., 2022). According to the well-recognized MSE guidelines (WHO, 2020), in this study, the variable of MSE was treated as a binary variable in the statistical analyses (0 = not meet [reporting 0–2 days], 1 = meet [reporting 3–7 days]).

### Covariates

Information on study participants’ sex (female/male), grade, race/ethnicity, eating behavior relative variables (drinking fruit juice and milk, eating fruit, green salad, potatoes, carrots, other vegetables, breakfast), and participating in physical activity, and watching television were measured by a self-reported questionnaire. These factors were treated as covariates in further statistical analysis (see Supplementary Table 1).

### Statistical analysis

Statistical Product and Service Solutions (SPSS) version 26.0 was used for all statistical analyses. The complex survey commands based on design were adopted to adjust the complex design of the YRBS survey sample. Population weights were included to adjust unequal selection possibilities. The complete case was performed to process the missing data (all the cases with missing data were removed). Descriptive analysis was applied to report the characteristics of the individual sample. Binary analyses of logistic regression were performed to check the relationship between meeting combinations of the MSE guidelines for obesity (yes or no), overweight (yes or no), depressive symptoms (yes or no), eating behavior (yes or no), physical activity at least 60 min per day 5 days or more days (yes or no), watched television three or more hours per day (yes or no), played video or computer games or used a computer three or more hours per day (yes or no), played on at least one sports team (yes or no). A generalized linear model was performed to predict relations between MSE guidelines and obesity, overweight, and depression. Statistical significance has been defined as *P* < 0.05.

## Results

Characteristics of the study sample are displayed in Table 1. The sample consisted of 50.3% girls (*n* = 6,885) and 48.6% boys (*n* = 6,641), and grade 10 was the most participants (27.2%). One in five participants reported zero days of MSE per week, followed by three (7.8%) and seven (7.7%) days. In total, 30.1% met the MSE recommendation. Around four in five adolescents who were overweight (*n* = 10,207, 74.6%) or obese (n = 10,345, 75.6%) failed to meet MSE guidelines, and there was more than one in three participants under depressive symptoms (*n* = 4,926, 36.0%).

**TABLE 1:** Sample characteristics of study participants.

		*n*	%	Weighted %	95% CI	
Sex	Girl	6,885	50.3	49.4	47.9	50.9
	Boy	6,641	48.6	50.6	49.1	52.1
	Missing	151	1.1			
Grade	9	3,637	26.6	26.6	25.4	28.0
	10	3,717	27.2	25.5	24.7	26.3
	11	3,322	24.3	24.3	23.2	25.4
	12	2,850	20.8	23.6	22.4	24.8
	Missing	151	1.1			
Race/ethnicity	White	6,668	48.8	51.2	46.4	56.0
	Black or African American	2,040	14.9	12.2	10.2	14.6
	Hispanic/Latino	3,038	22.2	26.1	21.8	30.9
	All other races	1,493	10.9	10.5	7.9	13.9
	Missing	438	3.2			
MSE days	0 days	2,568	18.8	29.7	27.6	32.0
	1 day	812	5.9	9.2	8.4	10.2
	2 days	973	7.1	11.6	10.8	12.5
	3 days	1,067	7.8	12.6	11.3	14.1
	4 days	763	5.6	9.1	8.4	9.9
	5 days	877	6.4	10.5	9.4	11.8
	6 days	361	2.6	4.4	3.8	5.1
	7 days	1,053	7.7	12.8	11.9	13.8
	Missing	5,203	38			
MSE guidelines	Meeting	4,121	30.1	49.5	47.6	51.3
	Not meeting	4,353	31.8	50.5	48.7	52.4
	Missing	5,203	38			
Overweight	Yes	1,933	14.1	16.1	14.9	17.5
	No	10,207	74.6	83.9	82.5	85.1
	Missing	1,537	11.2			
Obesity	Yes	1,795	13.1	15.5	13.8	17.3
	No	10,345	75.6	84.5	82.7	86.2
	Missing	1,537	11.2			
Depressive symptoms	Yes	4,926	36	36.7	35.1	38.3
	No	8,495	62.1	63.3	61.7	64.9
	Missing	256	1.9			

MSE, muscle-strengthening exercise; CI, confidence interval.

MSE guidelines: children and adolescents should amass three times per week; adults should amass two times per week.

Table 2 shows the results of multilevel logistic regression, and it can be found that there is a relationship between the potential correlations and meeting the MSE guidelines in the study sample. Participants who meet guidelines are more likely to be obese than those who are not (OR = 1.28, 95% CI = 1.06–1.55). The associations between MSE guidelines and overweight, depressive symptoms were no longer statistically significant in the adjusted model (OR = 0.86, 95% CI = 0.73–1.01: OR = 0.94, 95% CI = 0.83–1.06).

**TABLE 2:** Association between muscle strength exercise (MSE) guidelines and obesity, overweight, and depressive symptoms.

		OR	95% CI	
Obesity MSE guidelines	No	1.28	1.06	1.55
	Yes	REF		
Overweight MSE guidelines	No	0.86	0.73	1.01
	Yes	REF		
Depressive symptoms MSE guidelines	No	0.94	0.83	1.06
	Yes	REF		

MSE, muscle-strengthening exercise; CI, confidence interval.

MSE guidelines: children and adolescents should amass three times per week; adults should amass two times per week; The model controlled: sex (female/male), grade and race/ethnicity.

## Discussion

This study aimed to examine the association between meeting MSE guidelines and the prevalence of overweight or obesity and depression among American adolescents. Since few studies focused on this association, this study could provide a new perspective for preventing obesity among adolescents. The main findings indicated that regardless of sex, meeting the MSE guideline was associated with a lower level of obesity rather than overweight.

The present study revealed that meeting MSE recommendations were related to a lower probability of obesity. Even if limited population-based studies were concentrated on the association between meeting MSE guidelines and obesity, considerable experimental evidence proved that MSE positively impacted obesity among adolescents (Shaibi et al., 2006; Behm et al., 2008; Lee et al., 2012; Dias et al., 2015). To name but a few, one review study implied that moderate-intensity of MSE could reduce obesity and overweight among adolescents (Dietz et al., 2012). Another study showed that body fat reduction was significant after exposure to a 12-week MSE in obese adolescents (Dias et al., 2015). Beyond that, the body fat decreased significantly after accepting a 16-week resistance training program among male adolescents (Shaibi et al., 2006). One randomized controlled trial demonstrated that resistance exercise was correlated with the reduction of abdominal fat in obese male adolescents (Lee et al., 2012). Given the limitation of samples (Shaibi et al., 2006; Lee et al., 2012), it is necessary to delve into the association between MSE and obesity in both boys and girls in the future. Similar to one cross-sectional study on children and adolescents (Palacio-Agüero et al., 2020), our study also found that the MSE was inversely associated with obesity among American adolescents. However, MSE days were irrelevant to a lower level of obesity among American adolescents in the present study. According to the WHO recommendations, positive impacts will be possible when adolescents participate in muscle strength exercises at least 3 days a week (World Health Organization, 2020). Previous studies demonstrated that MSE could increase metabolic rate and/or total energy expenditure (Buzzachera et al., 2015). Hence, the MSE at least 3 days/week might be viewed as a benefit for a healthy weight, and an accumulated dose might be required for the positive effects of the MSE on low-level obesity among adolescents.

The MSE was not associated with overweight among adolescents in this population-based study, which was consistent with several studies. For example, the previous studies demonstrated that the MSE (e.g., weight training) had little effect on reducing overweight among adolescents (Atlantis et al., 2006). However, the included studies in this review implied that the weight training frequency was less than 3 days/week (Atlantis et al., 2006; Haykowsky et al., 2007; Caron et al., 2016), which might be a possible reason for the heterogeneity between this review and MSE guidelines. An evidence-based review proved that participating in resistance training programs did not impact adolescents’ weight and body composition (Malina, 2006). Due to the large heterogeneity in the measurement of the MSE in the previous studies and the lack of comparable cross-sectional studies, as well as adolescents in a period of rapid changes in the body, there are limited studies to identify the relationship between the MSE and overweight. In the future, more studies should focus on this topic.

The current study found no association between meeting the MSE guidelines and depression. Based on the results of this study, no significant association between meeting MSE guidelines and depressive symptoms was discovered. For instance, as Bennie et al. (2019) suggested, individuals who meet the guidelines for the MSE had lower risks of developing depression. A potential explanation of the current results was that the previous studies were mainly conducted among the adult population, and the findings are not generalizable to younger adolescents populations. Furthermore, it might be possible that the preventive effect of the MSE on depression is only significant for those with severe depressive symptoms. In a study conducted in Germany, the researchers recruited over 20 thousand respondents, proving a negative relationship between MSE levels and the occurrence of depression (Bennie et al., 2020b). Moreover, this correlation seems to become increasingly significant with participants who suffer from more severe depressive symptoms. As a result, further studies need to consider the severity of depression when investigating the relationship between physical activity level and depressive disorders.

### Practical implication

Our study has revealed that MSE is inversely associated with obesity among American adolescents, in contrast, no significant relationship has been identified between MSE and depression, which is worthy of further investigation. Overall, the finding implicates the role of strength training in the treatment of overweight. The fact is that adding resistance training to the workout routine can greatly preserve muscle mass and elevate metabolism to support weight reduction. However, contrary to the result of trustworthy scientific research, a large portion of people prefer aerobic activities instead of strength training as they suppose that it is only applicable to bodybuilders. This is quite a popular misunderstanding. From a clinical perspective, this study suggests that fitness coaches or doctors can add individualized strength training to obese people to improve muscle strength, improve exercise performance and complete more high-intensity fat-burning exercises. Further, the finding indicates that not only obese people but others with chronic health problems can work out to meet MSE guidelines since promoted strength and coordination brought by MSE are required for daily activities and benefit every one of all ages.

### Strengths and limitations

This study could contribute to this topic in the following aspects. On the one hand, we used a representative sample to delve into the association between the MSE guidelines and obesity and overweight. On the other hand, this study might be one of the limited ones focusing on adolescents.

Several limitations should be acknowledged. First and foremost, due to the limitation of cross-sectional investigation, the causal inferences between the MSE guidelines and obesity are not the scope of our study. Other factors like dietary, mental health, and aerobic physical activity should be considered in future studies. Besides, only adolescents were included in this study, but other population subgroups should be included in future studies. Finally, future large-scale interventions or longitudinal studies should further confirm the association between MSE guidelines and obesity, overweight and depressive symptoms in varied populations.

## Conclusion

The main finding of this study is that meeting MSE guidelines was positively associated with a lower level of obesity, but this association is not found between days of MSE and a lower prevalence of obesity among United States adolescents. Besides, no association was observed between meeting MSE guidelines and depression in United States adolescents.

## Data availability statement

The original contributions presented in this study are included in the article/Supplementary material, further inquiries can be directed to the corresponding authors.

## Ethics statement

Ethical review and approval were not required for the study on human participants in accordance with the local legislation and institutional requirements. Written informed consent to participate in this study was provided by the participants’ legal guardian/next of kin.

## Author contributions

JS: writing—original draft. JY and MG: formal analysis. XX, JY, and XZ: writing—review and editing. MG and XZ: supervision. JY: project administration. XZ and JS: funding acquisition. All authors read and agreed to the published version of the manuscript.

## Footnotes

1
https://www.cdc.gov/healthyyouth/data/yrbs/index.htm
