# Supplementary material for: Associations of muscle-strengthening exercise with overweight, obesity, and depressive symptoms in adolescents: Findings from 2019 Youth Risk Behavior Surveillance system
Source: Front Psychol. 2022 Sep 8;13:980076. doi: 10.3389/fpsyg.2022.980076 (PMC9495934; doi:10.3389/fpsyg.2022.980076)
Supplement: Supplementary file 1 [file Table_1.docx]

**Supplementary table 1: Demographic variables**

|  |  | **n** | **%** |  | **Weighted %** | **95%CI** | |
| --- | --- | --- | --- | --- | --- | --- | --- |
| **Did not drink fruit juice** |  |  |  |  |  |  |  |
|  | Yes | 3965 | 29.0 |  | 30.8 | 29.2 | 32.4 |
|  | No | 8627 | 63.1 |  | 69.2 | 67.6 | 70.8 |
|  | Missing | 1085 | 7.9 |  |  |  |  |
| **Did not eat fruit** |  |  |  |  |  |  |  |
|  | Yes | 1631 | 11.9 |  | 11.9 | 10.5 | 13.4 |
|  | No | 11255 | 82.3 |  | 88.1 | 86.6 | 89.5 |
|  | Missing | 791 | 5.8 |  |  |  |  |
| **Did not eat green salad** |  |  |  |  |  |  |  |
|  | Yes | 5101 | 37.3 |  | 41.9 | 39.3 | 44.6 |
|  | No | 6797 | 49.7 |  | 58.1 | 55.4 | 60.7 |
|  | Missing | 1779 | 13.0 |  |  |  |  |
| **Did not eat potatoes** |  |  |  |  |  |  |  |
|  | Yes | 4449 | 32.5 |  | 36.3 | 35.1 | 37.5 |
|  | No | 7450 | 54.5 |  | 63.7 | 62.5 | 64.9 |
|  | Missing | 1778 | 13.0 |  |  |  |  |
| **Did not eat carrots** |  |  |  |  |  |  |  |
|  | Yes | 6432 | 47.0 |  | 53.3 | 51.1 | 55.5 |
|  | No | 5445 | 39.8 |  | 46.7 | 44.5 | 48.9 |
|  | Missing | 1800 | 13.2 |  |  |  |  |
| **Did not eat other vegetables** |  |  |  |  |  |  |  |
|  | Yes | 2347 | 17.2 |  | 19.8 | 17.9 | 21.9 |
|  | No | 9500 | 69.5 |  | 80.2 | 78.1 | 82.1 |
|  | Missing | 1830 | 13.4 |  |  |  |  |
| **Did not drink milk** |  |  |  |  |  |  |  |
|  | Yes | 2919 | 21.3 |  | 30.6 | 29.0 | 32.3 |
|  | No | 6570 | 48.0 |  | 69.4 | 67.7 | 71.0 |
|  | Missing | 4188 | 30.6 |  |  |  |  |
| **Did not eat breakfast** |  |  |  |  |  |  |  |
|  | Yes | 1956 | 14.3 |  | 16.7 | 15.3 | 18.1 |
|  | No | 9637 | 70.5 |  | 83.3 | 81.9 | 84.7 |
|  | Missing | 2084 | 15.2 |  |  |  |  |
| **Were physically active at least 60 minutes per day on 5 or more days** |  |  |  |  |  |  |  |
|  | Yes | 5625 | 41.1 |  | 44.1 | 41.9 | 46.3 |
|  | No | 7595 | 55.5 |  | 55.9 | 53.7 | 58.1 |
|  | Missing | 457 | 3.3 |  |  |  |  |
| **Watched television 3 or more hours per day** |  |  |  |  |  |  |  |
|  | Yes | 2596 | 19.0 |  | 19.8 | 18.3 | 21.3 |
|  | No | 10200 | 74.6 |  | 80.2 | 78.7 | 81.7 |
|  | Missing | 881 | 6.4 |  |  |  |  |
| **Played video or computer games or used a computer 3 or more hours per day** |  |  |  |  |  |  |  |
|  | Yes | 5931 | 43.4 |  | 46.1 | 44.4 | 47.9 |
|  | No | 7246 | 53.0 |  | 53.9 | 52.1 | 55.6 |
|  | Missing | 500 | 3.7 |  |  |  |  |
| **Played on at least one sports team** |  |  |  |  |  |  |  |
|  | Yes | 5545 | 40.5 |  | 57.4 | 54.3 | 60.4 |
|  | No | 4242 | 31.0 |  | 42.6 | 39.6 | 45.7 |
|  | Missing | 3890 | 28.4 |  |  |  |  |
